# Perspectives on Universal Free School Meals Among Rural Middle and High School Students: A Mixed-Methods Study

**DOI:** 10.3390/nu18030540

**Published:** 2026-02-06

**Authors:** Ashley Kesack, Michele Polacsek, Alexis Schneider, Juliana F. W. Cohen

**Affiliations:** 1 Center for Health Innovation, Research, and Policy (CHIRP), Merrimack College, 315 Turnpike Street, North Andover, MA 01845, USA; kesackas@merrimack.edu; 2 School of Public and Planetary Health, University of New England, 716 Stevens Ave, Portland, ME 04103, USA; 3 College of Osteopathic Medicine, University of New England, 716 Stevens Ave, Portland, ME 04103, USA; 4 Department of Nutrition, Harvard T.H. Chan School of Public Health, 677 Huntington Ave, Boston, MA 02115, USA

**Keywords:** universal free school meals, rural, school meal participation, schools, students

## Abstract

**Background/Objectives**: School meals play an important role in ensuring reliable, healthy access to food, but there are many barriers to participation, especially for rural students. This study examined the perceived impact of UFSM policies and the perceptions of school meals among students in rural areas of Maine. **Methods**: This mixed-methods study included surveys with *n* = 61 middle and high school students and interviews with *n* = 11 middle and high school students between 2023 and 2024. The surveys and interviews focused on potential impacts of UFSM policies as well as general barriers and facilitators to participation in school meals. Quantitative surveys were analyzed using logistic regression, adjusting for demographic characteristics. Interviews were analyzed using principles of content analysis. **Results**: Students reported that UFSM had multiple benefits, and many students perceived that providing free school meals for all helped reduce the stigma associated with school meals, in part through increased participation. Students liked the variety of foods offered, but many did not perceive school meals, especially breakfast, to be “fresh” or “healthy”. Additionally, many students perceived there to be long lunch lines and insufficient time to eat their meals. **Conclusions**: While students perceived multiple benefits to UFSM, opportunities to further improve the quality of school meals, as well as policies to ensure sufficient lunch period lengths, may be important complements to increase participation and consumption of school meals among rural students.

## 1. Introduction

School meals have been found to improve child health outcomes and combat food insecurity, particularly among children from households with low income [1]. Approximately 28.6 million U.S. students eat school lunch, 14.5 million students eat school breakfast each day, and research shows that children typically get their healthiest meals at school [1,2,3]. Schools that participate in the National School Lunch Program and School Breakfast Program in the U.S. are required to serve healthy meals daily that include whole grains, a variety of vegetables, fruit, milk, and proteins, as well as limits on saturated fats, sodium, and added sugar [4]. However, there have historically been many barriers to eating school meals, including cost, complex school meal applications that low-income households must complete to receive free or reduced-price meals, meal taste and quality, the time allotted for eating, and stigma for both students and parents [5,6,7].

While there has been a substantial amount of research conducted on school meals, many studies have focused on urban or suburban schools [8,9]. However, rural schools represent about a third (34.9%) of schools participating in the National School Lunch Program, and school meal participation rates are higher on average in rural schools compared with those in urban areas [10,11,12,13]. However, rural areas often present unique challenges to healthy eating—both in schools and outside of school—including more limited access to and higher costs of healthy foods [14,15]. These conditions likely contribute to the poorer health outcomes of children and adolescents typically observed in these areas [10,11]. On average, 14.7% of people living in rural areas experience food insecurity, as compared to the national average of 12.8%, further highlighting the opportunity school meal programs offer children as a key social safety net that allows many students the opportunity to eat healthier foods at little to no additional cost [16]. In general, stigma and low satisfaction with school meals are common challenges to participating in school meals, and there is evidence that these concerns are more prevalent among students and parents in rural areas compared with those in urban or suburban areas [17,18]. In addition to the broader barriers to school meal participation, rural school food authorities (SFAs) also face many unique challenges, including higher foodservice costs and limited staff size and capacity, which can have a downstream impact on student experiences with school meals [10].

Universal free school meals (UFSM) policies—which enable all children to receive meals without charge—may help to address these challenges. Prior research has shown that UFSM policies can reduce barriers associated with school meals, like cost and stigma for students, and financial stability for school districts [19,20]. UFSM can result in increased participation, which can have a positive impact on students—including diet quality and food security for students and households more broadly—as well as on school finances [19,20]. Studies conducted among rural students in other countries with UFSM policies have found similar benefits among this population, but less is known about rural students in the U.S. [21,22,23]. While the federal government implemented a national UFSM policy to address rising food insecurity during the COVID-19 pandemic, this policy ended after the 2022–2023 school year (SY) [24]. However, several states—including California, Maine, Massachusetts, Nevada, and Vermont—subsequently passed state-level UFSM policies beginning during the 2023–2024 SY. While there have been a number of studies examining the perceived impact of these state-level policies on SFAs and parents [5,6,25,26,27], less is known about the perceptions and experiences of the students themselves, particularly in rural areas in the United States. Therefore, the aims of this mixed-methods study were to examine rural students’ perceived impact of UFSM policies on stigma and meal access due to meals being free for all students, and more broadly, their perceptions of school meals in rural areas of Maine.

## 2. Materials and Methods

This cross-sectional mixed-methods study included middle and high school students in rural regions of Maine in grades 6–12. Rural-Urban Commuting Area (RUCA) codes acquired from the USDA Economic Research Service were used to define rural locations (RUCA primary codes = 4–10). Participants were recruited using two independent research firms, and both parent consent and child assent were obtained. The research firms first contacted separate groups of parents (i.e., no overlap between participant pools) using proprietary consumer panel databases, which included socio-demographically diverse households with population characteristics of the state based on US Census data (2020 American Community Survey) [28]. The research firm asked parents to complete an online screener and survey via email or text message, which were administered through RedCap. The screener included questions about sociodemographic information for their household and consent for their child to participate in the study. Inclusion criteria (assessed through the screener) included having a child who was in middle or high school in a rural region of Maine, and attending a school that did not previously provide free school meals to all students prior to the state-level policy (e.g., through United States Department of Agriculture [USDA] provisions, such as the Community Eligibility Provision, which enables high poverty schools or districts to provide meals to all students within the district/school for free). Exclusion criteria included not having a middle or high school-aged child, if the child attended a private school, if they lived in a suburban or urban area in Maine, or if their child attended a school that previously provided free meals to all students prior to Maine’s UFSM law.

There was a 75% response rate among parents. After providing consent for their child to participate, the parent provided their child’s email address to share study information, obtain child assent, and send the study survey. Among students whose parents consented, 100% agreed to be in the study. The Institutional Review Board at the University of New England approved the study protocol (IRB # 0523-06, approved 15 May 2023).

A survey was developed for middle and high school students that focused on potential impacts of UFSM policies as well as general barriers and facilitators to participation in school meals. This survey was developed as part of a larger study examining the impact of UFSM across the U.S., among food service directors, parents, and students (including those in suburban and urban areas) [7,29]. Details about the survey have been previously published [29], but briefly, the survey was based on existing validated questionnaires [30,31,32] and was developed in collaboration with experts in the field of nutrition and school-based research, with the help of outside partners, including Full Plates Full Potential, an organization dedicated to combating childhood food insecurity in Maine. The survey was pilot tested among students in multiple states prior to administration. The final survey was administered through RedCap at two time points using two independent research firms, one during the summer and fall of 2023 and again in the spring of 2024. Students were provided a USD 15 incentive for the completion of the survey, which took approximately 20 min to complete.

Qualitative data was also collected to help contextualize the survey data and provide a more in-depth understanding of perceived impacts of UFSM, obtain a broader understanding of barriers and facilitators to participation in school meals, and understand student perception of school foods and free school meals. The research team developed question guides for the interviews based on the scientific literature related to UFSM and included input from experts in the field of school nutrition and anti-hunger organizations, as well as the Robert Wood Johnson Foundation’s Healthy Eating Research (HER) and Centers for Disease Control and Prevention’s (CDC) Nutrition and Obesity Policy Research and Evaluation Network (NOPREN) COVID-19 Qualitative Workgroup. The semi-structured interview guide enabled spontaneous follow-up questions and probes for clarifications and/or to expand on the discussion of relevant topics raised by participants. These interviews were conducted via Zoom (using a secure link) by the study research team among a random subsample of rural students from the survey data collection. Both parents and students agreed to the child being recorded, and the child’s oral assent was obtained prior to starting the interview. Each interview took approximately 30 min to complete. All interviews were recorded, transcribed, and analyzed for qualitative trends.

### Analysis

Descriptive statistics (frequencies, percentages) were calculated for the full sample of students. Logistic regression was used to compare survey results (examining the survey outcome of agree vs. disagree), examining differences by grade and gender (both variables simultaneously in the model). Statistical analyses were conducted using SAS statistical software (version 9.4, SAS Institute Inc., Cary, NC, USA) [33]. The qualitative analyses of student interviews relied on a grounded theory approach to understand the students’ perceptions of school meals and universal free school meal policies. First, the lead author (who identified as a white female from rural New England) reviewed the transcripts and developed codes and sub-codes based on key themes. The themes and codes were then reviewed and discussed with the research team. Subsequently, using immersion-crystallization methodology (an inductive, iterative process for identifying themes, categories, and patterns in qualitative data), the lead author formally coded the interviews and refined the codebook to further reflect the transcripts’ content (with coding further reviewed and discussed by the research team). A total of 12 primary code groups, 67 codes, and 10 sub-codes were created. The lead author coded the transcripts with the finalized codes using Excel^®^ version 14.0.0. Reports from the codes were then used to create themes, and from these, domains summarized the primary study outcomes. Key themes were consistently mentioned throughout the interviews, and no new themes emerged in the final interviews or analysis of the transcripts, suggesting saturation had been reached.

## 3. Results

### 3.1. Survey Data

#### 3.1.1. Survey Participant Demographics

A total of *n* = 61 students were recruited to complete surveys (100% of students who received parent consent), and *n* = 11 students completed interviews. Participant demographics are presented in Table 1. The grade level of the participants was evenly distributed between grades 6 and 12. Approximately half of the participants (49%) were girls, and the majority (90%) were Caucasian, reflective of the overall race/ethnicity within rural Maine more broadly. Each student attended a different school within Maine (representing 59% of the 103 non-CEP rural middle and high schools in Maine).

#### 3.1.2. School Breakfast: Participation and Student Perceptions

The majority of students (87%) reported that their school participated in the School Breakfast Program, whereas 6.5% reported that it was not available, and 6.5% reported that they were unsure whether their school participated. Additionally, 41% of students reported that their school provided grab-and-go/second-chance breakfast options. When asked how frequently they participated in school breakfast, roughly a quarter (23%) reported no participation, about a quarter (28%) reported 1–2 days/week, roughly 13% reported they participated 3–4 days/week, and about a quarter (23%) reported they participated daily (Table 1). When asked where they typically got breakfast on a school day (students were able to select more than one answer), over half (53%) reported getting breakfast at home and half (51%) reported getting breakfast from the school cafeteria (this may represent students who potentially consume breakfast at home and at school, since they could select more than one answer). In contrast, less than 4% of students reported obtaining breakfast off campus (from a restaurant, market, or store, or from a friend). Nearly 25% of students reported that they did not usually eat breakfast on school days.

When examining student perceptions, nearly two-thirds (62%) of the students reported that they arrived early enough to get school breakfast (Table 2). Just over half (51%) reported that they liked school breakfast, and over a third (38%) felt school breakfast was similar to what they ate for breakfast at home. However, about half (45%) did not feel that school breakfast was healthy, 58% of students thought they did not have enough time to eat breakfast at school, and 38% felt that school breakfast was not similar to what their family ate at home. Among these variables, there were no statistically significant differences by gender or grade level (middle vs. high school). However, when asked if they felt embarrassed to eat school breakfast, significantly more boys (23%) than girls (under 4%) reported that they were embarrassed to eat school breakfast (OR 10.8, 95% CI 1.1–103.8).

#### 3.1.3. School Lunch: Participation and Student Perceptions

When examining school lunch participation, 44% of students reported eating school lunch every school day (Table 1); a third ate school lunch 3–4 school days, 16% ate it 1–2 school days, and about 7% ate it on no days. The majority of students (84%) reported typically getting lunch from the school cafeteria (versus bringing lunch from home [7%], purchasing competitive foods at school for lunch [3%], purchasing lunch off campus [3%], or not typically eating lunch [3%]). Most students (58%) reported that they liked school lunch (Table 3), and half (50%) viewed school meals as healthy. Similarly, slightly under half (48%) reported they had sufficient time to eat lunch. Over two-thirds of students (69%) felt that the lunch lines were too long, with on average 52% of boys perceiving this to be an issue compared with 83% of girls (OR 0.21, 95% CI 0.06–0.73). The majority of students (70%) felt there were enough places to sit in the cafeteria. Approximately a fifth of students (22%) reported they were embarrassed to eat school lunch (or conversely, 78% were not embarrassed). Of note, 27% felt that school lunch was not similar to what their family ate at home. The difference between middle and high school students was statistically significant, with, on average, 34% of high school students reporting being embarrassed, compared with 7% of middle school students (OR 6.02, 95% CI 1.16–31.27). Lastly, when asked if students agreed with the statement “School meals are only for students whose families don’t have much money”, less than a fifth of students (15%) agreed with that statement.

### 3.2. Semi-Structured Interview Data

A total of 11 interviews were conducted. These interviews centered around 4 domains: (1) perceived benefits of UFSM, (2) factors that impact school breakfast and lunch participation, (3) perceptions of current school meals, and (4) students’ perceptions of changes that would support school meal participation. The major themes and illustrative quotations regarding the perceived benefits of UFSM and general perceptions of school meals more broadly are summarized in Table 4 and Table 5, respectively.

#### 3.2.1. Perceived Benefits of UFSM

First, students perceived that (UFSM) increased school meal participation, which normalized school meals and led to reductions in perceived stigma (Table 4). One female high school student described the impact: “It feels like a lot more people get the lunch now that it’s free and more accessible because even with the reduced prices, reduced meals, some people I know couldn’t afford that still to get lunch every day, so now that it’s free for everyone, a lot more people take advantage of that and get the lunch and use it”. A male high school student noted, “So usually when I eat school lunches I don’t really think much about it. There’s no stigma in our school about eating school lunches. I think everyone can feel comfortable with it”. Additionally, students discussed how appreciative they were of UFSMs, because they felt free school meals were important to ensure consistent access to healthy meals for all students. A female high school student shared the following: “I think it’s very important, because home lives, for all different people, are completely different, and I know there were times in my own life that I wouldn’t have been able to afford it. So schools giving people free lunches are sometimes the only meals kids will eat in a day. So it’s very important”. They also discussed how UFSMs reduced the stress experienced by students and their families. As one male middle school student explained, “So they can eat in school. So you don’t have to worry about coming home. At some houses, they don’t have that much food. Some students are kind of grateful that they have food at the school”. Similarly, a female high school student highlighted, “I’m very grateful. Before that even happened, I was on the free lunch list because I could not afford to pay every single day for school lunch. And I think a lot of students were feeling the same way. So I’m very grateful that we did this because I feel like it takes a lot of stress off about getting lunch”.

#### 3.2.2. Students’ General Perceptions of School Meals

When discussing school meals in general, students highlighted the convenience of both school breakfast and school lunch (Table 5). In addition to convenience, socialization was an important factor during school meals. One male high school student noted, “I’d say the major one is just socializing and just like getting a break and hanging out”. Variety was also highlighted as an important factor when deciding to eat school meals. As one female middle school student explained, “There’s options that are there every single day, like they’re like peanut butter and jelly sandwiches, and like salad, I guess, but then they have like a special thing every day, and I think that’s less fresh. But I’m glad that they have a lot of options”.

When asked about differences between school lunch and breakfast, students reported that breakfast is more “grab and go” and more “like a snack” as opposed to the full meal available for the school lunch. One female high school student explained, “Other than the portioning … they’re all about the same. I guess for breakfast they don’t serve as much of a big hot meal. It’s more just grab-and-go things other than sit down and have a meal”. Students also reported that school breakfast often contained mostly packaged items and lacked the fruits or vegetables available in the school lunches. Another female high school student shared: “School lunch, they at least try to put fruits and vegetables. Breakfast, there are no options for fruit and vegetables”.

Students perceived that foods in school meals were not always fresh and were often processed or packaged, and therefore, there were mixed opinions regarding whether school meals were perceived as fresh or healthy. One female high school student elaborated about lunch: “A lot of the time I eat a lot of salads and sandwiches. The lettuce is brown, shriveled, the veggies don’t taste the freshest. Usually we have apples that have a lot of bruises, bruised bananas. It’s not the freshest produce”. Similarly, when discussing breakfast, another female high school student noted that “There is really no freshness because a lot of it’s processed”. Conversely, one female high school student shared that “It’s definitely way more fresh than lunch”.

When asked about the differences between foods at home versus foods at school, students reported that foods at home were mostly made from scratch, while school meals were not. They did not feel that school meals tasted homemade. One female middle school student stated, “They’re really different, because at school usually they come in a package, and they just have to warm it up. And then at home, usually a meal is made”. While students seemed to like the variety of foods offered and cited this as a major reason for participation, they mentioned processed foods often and typically did not feel strongly that the meals were of good quality and taste, often describing them as average. One male middle school student explained: “They don’t taste like they were just made. They taste like they’ve been pre-made or packed”.

When discussing future needs, the same themes of a desire for fresher and healthier foods emerged as being important to students (in addition to a heartier, healthier breakfast). Students noted that they would like to see enhanced quality in the meals and fewer packaged items. One male middle school student noted that, for breakfast, “I would change the fact that they’re packaged so they’ve been made months prior and they’re only warmed up”. Similarly, when speaking about lunch, a female high school student highlighted, “More fresh ingredients would be good. A lot of the ingredients are not fresh at all. That’s one of the biggest things I’d say”.

## 4. Discussion

Overall, students reported that they frequently ate school lunch and felt that providing UFSM for all students helped reduce the stigma associated with school meals through increased participation. While students liked the variety of foods offered, most did not perceive school meals to be fresh or healthy, nor similar to what was consumed at home. There were statistically significant differences between boys and girls when asked if they felt embarrassed to eat school breakfast; 23% of boys reported that they were embarrassed to eat school breakfast, while just under 4% of girls reported this. The majority of students (78%) were not embarrassed to eat school lunch; however, this study found a statistically significant difference in embarrassment between middle and high school students, with, on average, 34% of high school students reporting being embarrassed, compared with 7% of middle school students.

In the state of Maine, over 113,000 students participate in the school lunch program; however, just under 60% participate in the breakfast program [2]. Nationally, in rural communities, there can be lengthy travel times between students’ homes and school, which results in limited time for students to participate in school breakfast [10]. Our study results mirrored national findings in that the majority of students reported that their school participated in the School Breakfast Program, but nearly a third of students reported they did not arrive at school early enough to participate in the breakfast program. Of note, 41% of students reported that their schools provided grab-and-go/second-chance breakfast options, which can help address some of the barriers to accessing school breakfast.

When examining embarrassment and stigma associated with school meals, boys were more likely to report embarrassment regarding eating school breakfast compared with girls. This may be due to girls skipping breakfast, as prior research has shown that female students are more likely to skip breakfast than males for weight-control reasons [34]. As for feelings of embarrassment around lunch, approximately a fifth of students reported they were embarrassed to eat school lunch, whereas the majority reported that they were not embarrassed to eat school lunch, which aligns with the theme of reduced stigma due to UFSM. Interestingly, there were some differences by grade level, with older students perceiving more embarrassment compared with younger students. This may be in part due to older students experiencing more years without a free school meal policy across all their years of school (due to the relative newness of Maine’s UFSM policy) and, therefore, may be more likely to retain feelings of embarrassment. Girls were more likely to report feelings of embarrassment at lunch than boys, which was a reversal from breakfast, which may be due in part to more girls participating in school lunch. Encouragingly, when asked if students agreed with the statement that “School meals are only for students whose families don’t have much money,” less than a fifth of students (15%) agreed with this statement. This study’s results align with past research that has shown that UFSMs reduce the stigma of school meals [5,6,25,26,27].

This study found that slightly under half of the students felt they had sufficient time to eat lunch. This may be in part due to lunch lines being too long, with 69% of students in the present study reporting this to be an issue. This also aligns with prior research documenting this issue with long lunch lines and insufficient time to eat [35,36,37,38]. The problem of long lines may be in part due to some of the unique challenges that rural schools may face when it comes to staffing. Specifically, rural schools offer lower compensation compared with other locales, which can impair recruitment and staff retention [10]. This could lead to staffing shortages, resulting in longer lines. These issues could partially be addressed through longer lunch periods.

Additionally, the majority of students in this study did not perceive the foods served at breakfast or lunch to be healthy or similar to what they ate at home. Based on the student interviews, this seemed to be in part due to students perceiving many school foods to be pre-made or pre-packaged, whereas foods at home were perceived as homemade.

While all the schools in this study participated in the National School Lunch Program, and thus served similar meal components daily (e.g., whole grains, vegetables, fruits, milk, proteins), there are not currently requirements regarding how the foods are prepared (e.g., scratch-cooked on site, frozen heat and serve, etc.) and whether or not they are highly processed. Of note, there is currently a growing interest in reducing ultra-processed foods in schools, which may help to address some of these negative perceptions around school meals [39]. However, additional resources will likely be required for schools to be able to prepare more scratch-cooked meals, especially in rural areas, which tend to have additional challenges with funding and staffing levels [40].

This study had a number of limitations. First, this study had a small sample size and was only conducted in rural areas in the state of Maine, which may limit generalizability to other rural areas. Additionally, the majority of students in the present study were white. While this is reflective of the demographics of Maine, future research should examine geographically, racially, and ethnically diverse student populations. Additionally, this study relied on student perceptions rather than measures such as school meal participation and consumption. The strengths of this study include its mixed-methods design, collecting both quantitative and qualitative data from rural students.

## 5. Conclusions

Overall, the UFSM policy was perceived as having multiple benefits for middle and high school students in rural areas of Maine. However, many students perceived there to be long lunch lines and insufficient time to eat their meals, which could be barriers to participation despite meals being free for all students. Interestingly, boys reported higher feelings of embarrassment around eating school breakfast, while girls felt more embarrassed when it came to eating school lunch. Middle school students felt less embarrassed eating school lunch than high school students. Students also had mixed views of school meals more broadly, with an emphasis on wanting more “healthy” and “fresh” items that were less processed. This study has important implications for improving school food services and the school meal experience for students, particularly in rural areas. Opportunities to further improve the quality of school meals, as well as policies to ensure sufficient lunch period lengths and interventions to improve breakfast participation, can be important complements to universal free school meal policies to ensure high consumption rates of school meals among rural students. Future research should examine breakfast after the bell strategies to increase school breakfast participation in rural areas, as well as effective strategies to improve student perceptions of the quality of school meals to better align them with healthy versions of foods consumed at home, where feasible. In particular, research should examine both the strategies and resources needed to reduce pre-packaged, ultra-processed foods in schools and replace them with more scratch-cooked foods in rural areas where limited resources and staff could pose significant barriers.

## Figures and Tables

**Table 1:** Survey participant characteristics of middle and high school students in rural regions of Maine (*n* = 61).

Student Characteristics	
Grade	*n* (%)
6	10 (16.4%)
7	8 (13.1%)
8	10 (16.4%)
9	11 (18%)
10	7 (11.5%)
11	11 (18%)
12	4 (6.6%)
Gender	
Girl	29 (47.5%)
Boy	29 (47.5%)
Other	3 (4.9%)
Student race and ethnicity	
White	55 (90.2%)
Other race/multiracial	3 (4.9%)
American Indian or Alaska Native	2 (3.3%)
Hispanic or Latinx	1 (1.6%)
Frequency of school breakfast participation	
1–2 school days	17 (27.9%)
Every school day	14 (23%)
No days	14 (23%)
3–4 school days	8 (13.1%)
No response	8 (13.1%)
Frequency of school lunch participation	
Every school day (Monday–Friday)	27 (44.3%)
3–4 school days	20 (32.8%)
1–2 school days	10 (16.4%)
No days	4 (6.6%)
Where do you typically get breakfast on a school day ^1,2^	
At home	28 (52.8%)
From the school cafeteria	27 (50.9%)
I don’t usually eat breakfast on school days	13 (24.5%)
Off campus (from a restaurant, market, or store)	2 (3.8%)
I get breakfast from a friend	2 (3.8%)
From a school vending machine, school snack bar, a la carte line or single items purchased in the cafeteria	1 (1.9%)
Where do you typically get lunch on a school day ^1^	
From the school cafeteria or school food service window	52 (85.2%)
Bring lunch from home	13 (21.3%)
From a school vending machine, school snack bar, a la carte line or single items purchased in the cafeteria	6 (9.8%)
I typically skip lunch on school days	6 (9.8%)
Off campus (from a restaurant, market, or store)	4 (6.6%)
I get lunch from a friend	1 (1.6%)

^1^ Students were able to select more than one answer. ^2^ *n* = 8 students did not report breakfast participation.

**Table 2:** Perceptions of school breakfast among middle and high school students in rural regions of Maine (*n* = 61).

Survey Question	Overall	High School (*n* = 33)	Middle School (*n* = 28)			Male (*n* = 29)	Female (*n* = 29)		
	% Agree	% Agree		OR	95% CI	% Agree		OR	95% CI
I arrive at school early enough to get a school breakfast	62.26	55.17	70.83	0.70	0.20–2.00	69.23	53.85	1.70	0.60–5.00
I usually like school breakfast	50.94	55.17	45.83	1.50	0.52–4.34	50.00	50.00	1.16	0.40–3.36
School breakfast is healthy	45.28	41.38	50.00	0.79	0.27–2.31	42.31	50.00	0.76	0.26–2.21
I have enough time to eat breakfast at school	41.51	44.83	37.50	1.32	0.44–3.97	38.46	42.31	0.97	0.32–2.89
School breakfast is similar to what my family eats at home	37.74	34.48	41.67	0.92	0.30–2.80	42.31	34.62	1.40	0.46–4.25
I feel embarrassed to eat school breakfast	13.21	17.24	8.33	4.2	0.70–26.50	23.08	3.85	**10.80**	1.10–103.80

Bold denotes statistically significant (*p* < 0.05).

**Table 3:** Perceptions of school lunch and meals of middle and high school students in rural regions of Maine (*n* = 61).

Survey Question	Overall	High School (*n* = 33)	Middle School (*n* = 28)			Male (*n* = 29)	Female (*n* = 29)		
	% Agree	% Agree		OR	95% CI	% Agree		OR	95% CI
I usually like school lunch	58.33	56.25	60.71	0.67	0.23–1.93	57.14	56.67	0.86	0.30–2.49
I get tired of the same foods at school lunch	56.67	56.25	57.14	0.89	0.31–2.57	57.14	56.67	0.92	0.32–2.64
School lunch is healthy	50.00	46.88	53.57	0.78	0.27–2.24	57.14	43.33	1.53	0.53–4.38
School lunch is healthier than foods I bring from home or can buy near school	26.67	28.13	25.00	1.25	0.38–4.10	28.57	26.67	1.10	0.40–3.58
I have enough time to eat lunch at school	48.33	46.88	50.00	0.88	0.31–2.52	53.57	43.33	1.36	0.48–3.90
The lunch lines in the school cafeteria are too long	68.85	72.73	64.29	0.99	0.30–3.23	51.72	83.33	**0.21**	0.06–0.73
School lunch is similar to what my family eats at home	26.67	28.13	25.00	1.10	0.33–3.68	28.57	23.33	1.28	0.38–4.26
I feel embarrassed to eat school lunch	21.67	34.38	7.14	**6.02**	1.16–31.27	10.71	33.33	0.29	0.07–1.27
There are enough places to sit in the cafeteria or the eating area	70.49	66.67	75.00	0.88	0.27–2.93	82.76	60.00	3.12	0.91–10.71
School meals are only for students whose families do not have much money	14.75	18.18	10.71	1.91	0.39–9.34	17.24	10.00	2.17	0.45–10.57

Bold denotes statistically significant (*p* < 0.05).

**Table 4:** Domains, themes, and illustrative quotes regarding Universal Free School Meals from middle and high school students in rural regions of Maine (*n* = 11).

Sub-Theme	Illustrative Quotes
Domain: Perceived Benefits of UFSM Reported by Students	
UFSM participation	“It feels like a lot more people get the lunch now that it’s free and more accessible because even with the reduced prices, reduced meals, some people I know could not afford that still to get lunch every day, so now that it’s free for everyone, a lot more people take advantage of that and get the lunch and use it.”—Female high school student “Because it’s free. They’re hungry. They don’t want to have to pay for it. They don’t have to worry about paying for it.”—Male high school student
Stigma	“I think now that my friends go up, it’s making me feel comfortable also going up. And last year my friends didn’t go up, we all just kind of sat and talked.”—Female high school student “I think that’s pretty good because then everyone has an option. And if people can’t afford normal lunch, and they have that, they have that option. And if it was only for, like certain people, they could feel more embarrassed, and because it is free for everyone, I guess some people feel more comfortable.”—Female middle school student “So usually when I eat school lunches I don’t really think much about it. There’s no stigma in our school about eating school lunches. I think everyone can feel comfortable with it.”—Male high school student
Stress	“I’m very grateful. Before that even happened, I was on the free lunch list because I could not afford to pay every single day for school lunch. And I think a lot of students were feeling the same way. So I’m very grateful that we did this because I feel like it takes a lot of stress off about getting lunch.”—Female high school student “So they can eat in school. So you don’t have to worry about coming home. At some houses, they don’t have that much food. Some students are kind of grateful that they have food at the school.”—Male middle school student
Access to healthy meals	“I think it’s very important, because home lives, for all different people, are completely different, and I know there were times in my own life that I wouldn’t have been able to afford it. So schools giving people free lunches are sometimes the only meals kids will eat in a day. So it’s very important.”—Female high school student “I think we’re there [in school] for many hours a day. You’re supposed to feed your body every three to five hours. And I think offering healthy, nutritious lunches for us is a mandatory thing.”—Female high school student “Because some people may not have the money or may be struggling with something like that and it’s really helpful for them. They still get a good meal during the day.”—Male high school student

**Table 5:** Domains, themes, and illustrative quotes regarding School Meals Overall from middle and high school students in rural regions of Maine (*n* = 11).

Sub-Themes	Illustrative Quotes
Domain: Participation in School Meals	
Reasons for eating school lunch: Students cite many reasons for eating school lunch, including convenience and socialization	“I guess just having my friends with me and then seeing them eat the salads and the sandwiches and being like, “Those look pretty good.”—Female high school student “We don’t have time to pack lunch, do we? In the morning, it’s also just easier. It’s more convenient.”—Male middle school student “Well, for me, I think for most, it’s probably getting up too late or you don’t have the time to make it the day before or you don’t even have food in general at home. So, there’s tons of reasons.”—Male middle school student “I’d say the major one is just socializing and just like getting a break and hanging out.”—Male high school student
Reasons for eating school breakfast: Students eat school breakfast because of the availability, the multiple options available, and ease of getting it at school	“Because it’s good for you just to make sure that you are getting your food and not starving, that you’re nice and full throughout the day.”—Male high school student “What I like the best about school breakfast is the more choices there are.”—Male middle school student “I like that it’s available and I know it’s available. So, if I need to, I can get it.”—Male middle school student
Domain: Perception of School Meals	
Differences between foods at home and foods at school: Students mention meals at home are usually cooked from scratch, while foods at schools are typically more processed	“They’re really different, because at school usually they come in a package, and they just have to warm it up. And then at home, usually a meal is made.”—Female middle school student “It is very different, because when I eat dinner with my family, I know it’s good and it definitely feels better to eat than the school lunch.”—Male middle school student “Really different. Most of the food at home is homemade. I don’t know how they make it, but it doesn’t taste like homemade.”—Male middle school student
Differences between school lunch and breakfast: Students cite differences between breakfast and lunch, specifically more packaged snack-like foods for breakfast vs. a full meal like lunch	“School lunch, they at least try to put fruits and vegetables. Breakfast, there are no options for fruit and vegetables.”—Female high school student “Other than the portioning, I don’t think so. They’re all about the same. I guess for breakfast they don’t serve as much of a big hot meal. It’s more just grab-and-go things other than sit down and have a meal.”—Female high school student “I think they’re really good. Definitely better than lunch. You get, I think, 6 choices, which is a lot.”—Male middle school student
Freshness of school lunch: Opinions are mixed about the freshness of foods in school lunch, but students mention processed, packaged, and frozen foods contribute to their negative opinion of freshness	“More fresh ingredients would be good. A lot of the ingredients are not fresh at all.”—Male middle school student “It’s all very fresh, like meat, vegetables, and bread and stuff.”—Male high school student “A lot of the time I eat a lot of salads and sandwiches. The lettuce is brown, shriveled, the veggies don’t taste the freshest. Usually we have apples that have a lot of bruises, bruised bananas. It’s not the freshest produce.”—Female high school student
Healthfulness of school lunch: Students feel it is important for meals to be healthy, but find the healthfulness of school lunch to be lacking	“Oh, they’re not very healthy, because I feel that there’s a lot of like fast food sort of thing. There’s like pizza and like chicken nuggets and like cinnamon bar things, and it’s not very healthy.”—Female middle school student “Yeah, everything feels like it’s unhealthy and not freshly made.”—Male middle school student “It’s pretty important to me if we’re eating it every day. Like I said, not many people bring home lunch. So it means they’re eating that maybe five days a week. That’s pretty important if it’s healthy.”—Male middle school student
Quality and taste of school lunch: Perceptions of meal quality and taste were mixed, with most rating school lunch as average	“They don’t taste like they were just made. They taste like they’ve been pre-made or packed.”—Male middle school student “Meh. It’s average.”—Male middle school student “It’s like in between good and not so, not really good tasting. Mostly good.”—Male high school student “I’m not sure if this is just like a state regulation or anything but a lot of the time there’ll be no sort of seasoning. And sometimes it’ll be undercooked or overcooked at the same time. Definitely not the best. But it’s about what you’d expect for school lunch.”—Female high school student “Overall, it’s pretty good. There’s a couple things that don’t taste the same as buying it from the store, but overall they’re pretty good.”—Female high school student
Variety of school lunch: Students cite having many options for lunch and the wide variety of foods as a main reason why they eat school lunch	“There’s options that are there every single day, like they’re like peanut butter and jelly sandwiches, and like salad, I guess, but then they have like a special thing every day, and I think that’s less fresh. But I’m glad that they have a lot of options.”—Female middle school student “I feel like there’s a wide variety that can fit almost everyone.”—Male high school student “I definitely feel like high school food is better because you get more options.”—Female high school student
Quality and freshness of school breakfast: Students have mixed reviews about the quality and freshness of breakfast	“There is really no freshness because a lot of it’s processed.”—Female high school student “The breakfast, it’s amazing. Biting into that donut stick, it’s fresh and the glaze on it is amazing. It’s like you can’t just have one.”—Male high school student “It’s definitely way more fresh than lunch.”—Female high school student “Yeah, there’s no quality or there’s no freshness, but taste, it’s the same as lunch, just in the middle. It’s not good, it’s not bad. It’s just something you can eat.”—Male middle school student
Domain: Future Needs	
Changes to breakfast program: Students suggest healthier, heartier options for breakfast	“I would change the fact that they’re packaged so they’ve been made months prior and they’re only warmed up.”—Male middle school student “I don’t mind it because it’s like a snack. It’s not like a full meal. So yeah, they could maybe add cereal or something else, maybe waffles or something. Add more options.”—Male high school student “Healthier stuff.”—Female high school student
Changes to lunch program: There is room for improvement in school lunch meals	“I feel like they have all the things, but their water costs money. And that’s really weird to me, because you need water. And you can have chocolate milk and juice for free. And I don’t really like that, because if you just don’t bring a water bottle, I feel like it’s nice to have water too.”—Female middle school student “More fresh ingredients would be good. A lot of the ingredients are not fresh at all. That’s one of the biggest things I’d say.”—Female high school student
Ideal school lunch: Students describe their ideal school lunch	“It would be like a restaurant, but you get to pick. You choose what you want and boom, that’s your lunch. It’s basically what our school lunch is right now. It’s like a little restaurant.”—Male high school student “I like most of the foods they serve just maybe slightly more quality, like freshness to them, like a little bit more quality to some.”—Male high school student “I think if I were to get a lunch, it would definitely be high quality and made so that it feels healthy and not junky.”—Male middle school student

## Data Availability

The original contributions presented in this study are included in the article. Further inquiries can be directed to the corresponding author.

## Abbreviations

The following abbreviations are used in this manuscript:

SY	School Year
USDA	United States Department of Agriculture
UFSM	Universal Free School Meals

## References

[1] Au L.E., Gurzo K., Gosliner W., Webb K.L., Crawford P.B., Ritchie L.D. (2018). Eating school meals daily is associated with healthier dietary intakes: The Healthy Communities Study. J. Acad. Nutr. Diet..

[2] United States Department of Agriculture (2024). Child Nutrition Tables.

[3] Liu J., Micha R., Li Y., Mozaffarian D. (2021). Trends in food sources and diet quality among US children and adults, 2003–2018. JAMA Netw. Open.

[4] United States Department of Agriculture (2024). Final Rule—Child Nutrition Programs: Meal Patterns Consistent with the 2020–2025 DGAs.

[5] Zuercher M.D., Cohen J.F., Ohri-Vachaspati P., Hecht C.A., Hecht K., Polacsek M., Olarte D.A., Read M., Patel A.I., Schwartz M.B. (2024). Parent perceptions of school meals and how perceptions differ by race and ethnicity. Health Aff. Sch..

[6] Cohen J.F., Chapman L.E., Olarte D.A., Hecht C.A., Hecht K., Minc L., Ohri-Vachaspati P., Orta-Aleman D., Patel A.I., Polacsek M. (2024). Perceived Influence of a State-Level Universal Free School Meal Policy on Households with Varying Income Levels: An Analysis of Parental Perspectives. J. Acad. Nutr. Diet..

[7] Orta-Aleman D., Zuercher M.D., Bacon K.A., Chelius C., Hecht C.E., Hecht K., Ritchie L.D., Cohen J.F., Gosliner W. (2024). Students’ Perspectives on the Benefits and Challenges of Universal School Meals Related to Food Accessibility, Stigma, Participation, and Waste. J. Nutr. Educ. Behav..

[8] Hecht A.A., Olarte D.A., McLoughlin G.M., Cohen J.F. (2023). Strategies to Increase Student Participation in School Meals in the United States: A Systematic Review. J. Acad. Nutr. Diet..

[9] Cohen J.F., Hecht A.A., Hager E.R., Turner L., Burkholder K., Schwartz M.B. (2021). Strategies to improve school meal consumption: A systematic review. Nutrients.

[10] Cohen J., Turner L., Schwartz M. (2021). Rural Schools: Challenges and Opportunities for School Meal Programs.

[11] Asada Y., Mitric S., Chriqui J.F. (2020). Addressing equity in rural schools: Opportunities and challenges for school meal standards implementation. J. Sch. Health.

[12] Johnson L.D., Mitchel A.L., Rotherham A.J. (2014). Federal Education Policy in Rural America.

[13] Forrestal S., Cabili C., Dotter D., Logan C.W., Connor P., Boyle M., Enver A., Nissar H. (2019). School Nutrition and Meal Cost Study Final Report Volume 1: School Meal Program Operations and School Nutrition Environments.

[14] Liese A.D., Weis K.E., Pluto D., Smith E., Lawson A. (2007). Food store types, availability, and cost of foods in a rural environment. J. Am. Diet. Assoc..

[15] Steves A., Cho C., Metin Ç., Kong X., Boland M. (2021). The Food Retail Landscape Across Rural America.

[16] Rabbitt M.P., Hales L.J., Burke M.P., Coleman-Jensen A. (2023). Household Food Security in the United States in 2022.

[17] Fox M.K., Gearan E., Cabili C., Dotter D., Niland K., Washburn L., Paxton N., Olsho L., LeClair L., Tran V. (2019). School Nutrition and Meal Cost Study Final Report Volume 4: Student Participation, Satisfaction, Plate Waste, and Dietary Intakes.

[18] Meier C.L., Brady P., Askelson N., Ryan G., Delger P., Scheidel C. (2022). What do parents think about school meals? An exploratory study of rural middle school parents’ perceptions. J. Sch. Nurs..

[19] Cohen J.F., Hecht A.A., McLoughlin G.M., Turner L., Schwartz M.B. (2021). Universal school meals and associations with student participation, attendance, academic performance, diet quality, food security, and body mass index: A systematic review. Nutrients.

[20] Spill M.K., Trivedi R., Thoerig R.C., Balalian A.A., Schwartz M.B., Gundersen C., Odoms-Young A., Racine E.F., Foster M.J., Davis J.S. (2024). Universal free school meals and school and student outcomes: A systematic review. JAMA Netw. Open.

[21] Illøkken K.E., Johannessen B., Barker M.E., Hardy-Johnson P., Øverby N.C., Vik F.N. (2021). Free school meals as an opportunity to target social equality, healthy eating, and school functioning: Experiences from students and teachers in Norway. Food Nutr. Res..

[22] Afridi F. (2011). The impact of school meals on school participation: Evidence from rural India. J. Dev. Stud..

[23] Heim G., Thuestad R.O., Molin M., Brevik A. (2022). Free school meal improves educational health and the learning environment in a small municipality in Norway. Nutrients.

[24] United States Department of Agriculture FNS Responds to COVID-19. https://fns-prod.azureedge.us/sites/default/files/resource-files/cnp-operations-covid19-july2020-dec2021-summary.pdf.

[25] Chapman L.E., Gosliner W., Olarte D.A., Ritchie L.D., Schwartz M.B., Polacsek M., Hecht C.E., Hecht K., Turner L., Cohen J. (2024). Universal School Meals During the Pandemic: A Mixed Methods Analysis of Parent Perceptions from California and Maine. J. Acad. Nutr. Diet..

[26] Cohen J.F., Polacsek M., Hecht C.E., Hecht K., Read M., Olarte D.A., Patel A.I., Schwartz M.B., Turner L., Zuercher M. (2022). Implementation of universal school meals during COVID-19 and beyond: Challenges and benefits for school meals programs in Maine. Nutrients.

[27] Zuercher M.D., Cohen J.F., Hecht C.E., Hecht K., Ritchie L.D., Gosliner W. (2022). Providing school meals to all students free of charge during the COVID-19 pandemic and beyond: Challenges and benefits reported by school foodservice professionals in California. Nutrients.

[28] US Census Bureau (2020). American Community Survey (ACS), 2020.

[29] Orta-Aleman D., Zuercher M.D., Chapman L.E., Schwartz M.B., French C.D., Patel A.I., Ritchie L., Cohen J., Gosliner W. (2026). Universal School Meal Policies and Perceived Stigma: Quantitative Evidence From Eight US States. J. Sch. Health.

[30] United States Department of Agriculture (2019). School Nutrition and Meal Cost Study—Data Collection Instruments.

[31] Smith S., Cunningham-Sabo L., Auld G. (2015). Satisfaction of middle school lunch program participants and non-participants with the school lunch experience. J. Child Nutr. Manag..

[32] Machado S.S., Ritchie L.D., Thompson H.R., Madsen K.A. (2020). The Impact of a Multi-Pronged Intervention on Students’ Perceptions of School Lunch Quality and Convenience and Self-Reported Fruit and Vegetable Consumption. Int. J. Environ. Res. Public Health.

[33] (2023). SAS Statistical Software.

[34] Hearst M.O., Shanafelt A., Wang Q., Leduc R., Nanney M.S. (2016). Barriers, benefits, and behaviors related to breakfast consumption among rural adolescents. J. Sch. Health.

[35] Sharma A., Moon J., Bailey-Davis L., Conklin M. (2017). Food choices and service evaluation under time constraints: The school lunch environment. Int. J. Contemp. Hosp. Manag..

[36] Thompson H.R., Gosliner W., Ritchie L., Wobbekind K., Reed A.L., O’Keefe O., Madsen K.A. (2020). The impact of a multipronged intervention to increase school lunch participation among secondary school students in an urban public school district. Child. Obes..

[37] Berggren L., Olsson C., Talvia S., Hörnell A., Rönnlund M., Waling M. (2020). The lived experiences of school lunch: An empathy-based study with children in Sweden. Child. Geogr..

[38] Mancino L., Guthrie J.F. (2009). When nudging in the lunch line might be a good thing. Amber Waves: The Economics of Food, Farming, Natural Resources, and Rural America.

[39] Cohen J.F., Chapman L.E., Schwartz M.B. (2025). The implications of the Make Our Children Healthy Again report for child health and obesity in the US. BMJ.

[40] Gombi Vaca M.F., Lyerly R., Cohen J.F.W., Schwartz M. (2025). Ultra-Processed Foods in School Meals: Challenges and Opportunities.

